# Historic changes in length distributions of three Baltic cod (*Gadus morhua*) stocks: Evidence of growth retardation

**DOI:** 10.1002/ece3.3173

**Published:** 2017-06-28

**Authors:** Henrik Svedäng, Sara Hornborg

**Affiliations:** ^1^ Department of Aquatic Resources Institute of Marine Research Swedish University of Agricultural Sciences Lysekil Sweden; ^2^ RISE – Research Institutes of Sweden, Agrifood and Bioscience, Sustainable Food Production Gothenburg Sweden; ^3^ Present address: Swedish Institute for the Marine Environment (SIME) Gothenburg Swedenand; ^4^ Present address: Baltic Sea Centre Stockholm University Stockholm Sweden

**Keywords:** assessment, Baltic sea, growth indices, selectivity, size spectrum, stock productivity

## Abstract

Understanding how combinations of fishing effort and selectivity affect productivity is central to fisheries research. We investigate the roles of fishing regulation in comparison with ecosystem status for Baltic Sea cod stock productivity, growth performance, and population stability. This case study is interesting because three cod populations with different exploitation patterns and stock status are located in three adjacent but partially, ecologically different areas. In assessing stock status, growth, and productivity, we use survey information and rather basic stock parameters without relying on age readings. Because there is an urgent interest of better understanding of the current development of the Eastern Baltic cod stock, we argue that our approach represents partly a novel way of interpreting monitoring information together with catch data in a simplified yet more informative way. Our study reports how the Eastern and Western Baltic cod have gone toward more truncated size structures between 1991 and 2016, in particular for the Eastern Baltic cod, whereas the Öresund cod show no trend. We suggest that selective fishing may disrupt fish population dynamic stability and that lower natural productivity might amplify the effects of selective fishing. In support of earlier findings on a density‐dependent growth of Eastern Baltic cod, management is advised to acknowledge that sustainable exploitation levels for Eastern Baltic cod are much more limited than perceived in regular assessments. Of more general importance, our results emphasize the need to embrace a more realistic view on what ecosystems can produce regarding tractable fish biomass to facilitate a more ecosystem‐based fisheries management.

## 1 INTRODUCTION

### 1.1 Population dynamic stability and fishing

Understanding how combinations of fishing effort and selectivity affect productivity is central to fisheries research (Worm et al., 2009). Fluctuating fishing pressure may lead to amplifying temporal variability in fish abundance (Jonzén, Ripa, & Lundberg, 2002). Anderson et al. (2008) noted, however, that fishing in itself may give rise to fundamentally unstable population dynamics; a temporal instability arises as the stocks become increasingly age truncated and juvenescent, while older and bigger fish are wiped out due to fishing. This truncation in age and size is suggested to lead to higher somatic population growth, which in turn, enhances the intrinsic rate of population increase, *r*. The higher somatic population growth is seen as the principal driver of the variability in *r*, ultimately caused by competitive release and decreased cannibalism. In this study, we further investigate whether the opposite pattern can occur that truncated size structures may reduce stock productivity. We hence argue that selective fishing could affect *r* by limiting growth in biomass. This decline in growth is ultimately linked to increased intraspecific competition, that is, density‐dependent effects, as smaller and intermediate size classes, become more crowded. Lowered natural mortality due to reduced cannibalism from larger size classes may further exacerbate competition among smaller size classes.

The results are important in times of managing for fish stock recoveries. Fish abundances may increase from successful management efforts to curtail fishing mortality for over‐exploited stocks. When also gear selectivity (mesh sizes) is simultaneously increased, with the intention to reduce fishing mortality on smaller fish when fishers target for larger individuals of higher commercial value, there is a risk of inducing density‐dependent growth; as recruitment improves, the number of fish in size classes below length at first catch, *L*_c_, is increased (Beverton & Holt, 1957). This may lead to food limitations for fish in certain size classes. In fact, density‐dependent individual growth is a wide‐spread phenomenon and prime factor regulating stock sizes (Lorenzen & Enberg, 2002; Rose et al., 2001).

Furthermore, fishing‐induced changes in individual growth could potentially affect age at maturity, recruitment, and mortality rates, and, thereby also stock‐recruitment relationships (Beverton & Holt, 1959; Shelton et al., 2006). Such changes in individual growth may thus destabilize fish population dynamics as growth in population biomass and productivity may become essentially altered due to selective fishing, possibly reinforced by environmental forcing. This process could eventually lead to a series of fisheries‐induced boom‐and‐burst events, causing local extirpations (Anderson et al., 2008).

### 1.2 State of cod populations and fisheries in the Baltic Sea

**Figure 1:**
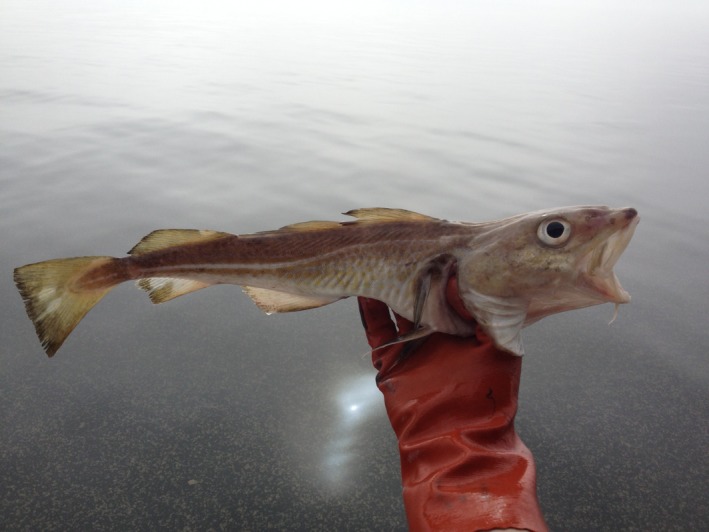
One of the study objects: the Eastern Baltic cod (© Peter Ljungberg)

**Figure 2:**
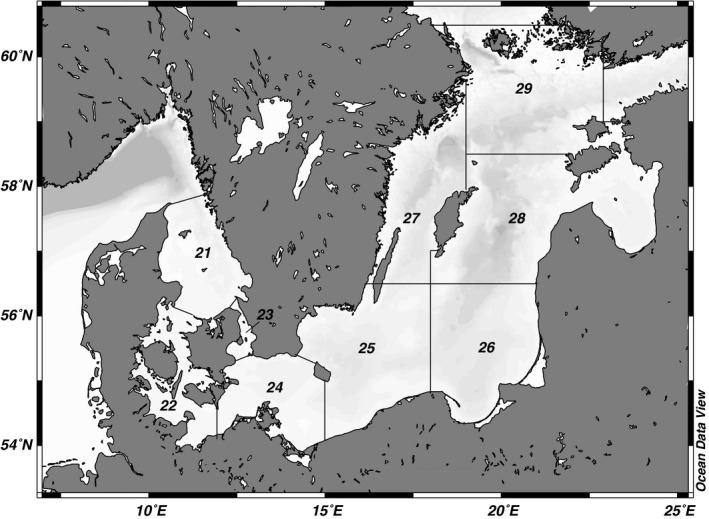
The Baltic Sea. The borders and numbers of the ICES subdivisions (SD) are as defined by the International Council for the Exploration of the Sea (www.ices.dk)

An illustrative case of selectivity‐induced instability is provided by the Eastern Baltic cod (*Gadus morhua*; Figure 1; Svedäng & Hornborg, 2014). The stock is presently restrained in an unproductive state, due to a noticeable decline in individual growth rates (ICES, 2015), or at least in condition factor (Casini et al., 2016). This poor nutritional status of the Eastern Baltic cod stock has been correlated to density‐dependent limitations of food (Eero et al., 2012). It is also suggested that oxygen deficits in the deeper parts of the Baltic Sea may hamper the production of benthic feed available for the cod stock (Casini et al., 2016). Sprat (*Sprattus sprattus*), which dominate cod diet, is still broadly abundant in the northern Baltic while it has become increasingly scarce in the southern part of the sea, where the cod stock is presently more or less confined to (Figure 2, subdivision (SD) 25; Eero et al., 2012).

Selective fishing may have reinforced food limitation for the Eastern Baltic cod stock. The abundance of small‐sized cod has increased as gear selectivity has gradually increased toward larger size classes over the last 20 years. Meanwhile, a steady or increasing fishing pressure on bigger cod has caused a severe decline of fish in larger size groups (Feekings, Lewy, & Madsen, 2013; Svedäng & Hornborg, 2014). Reduced abundance of large cod may also induce decreased cannibalism (Möllmann et al., 2014), further reducing natural mortality *M* in smaller size groups. As the smaller size classes become increasingly crowded, growth (measured by the growth parameter *L*_∞_ in the von Bertalanffy growth equation; cf. Beverton & Holt, 1957) declines. At stable levels of recruitment, or at high recruitment events, this process can be assumed to be self‐reinforced: As growth drops, the number of fish will inevitably increase in nonfishable size groups, slowing down their transition through intermediary size classes.

In support of such a process, Svedäng & Hornborg, 2014 found a negative relationship between growth (*L*_∞_) and population size at age 2, indicating that growth is presently density dependent in Eastern Baltic cod. However, the status of the Eastern Baltic cod stock is considered to be uncertain, caused by increasing difficulties in aging (Eero et al., 2015), and the stock is regarded as data poor since 2014 (ICES, 2014). Albeit condition has declined, it is suggested that a reduction in growth need be evidenced (Casini et al., 2016).

### 1.3 The studied fish stocks

Naturally, a better understanding of exploited fish population dynamics and their relationship with fishing selectivity and effort is needed to develop ecosystem‐based fisheries management (EBFM; e.g., Long, Charles, & Stephenson, 2015). In this sense, fisheries for Baltic cod can be viewed as a particularly interesting case study. It includes three areas harboring different stocks: the Öresund (SD23), west of Bornholm, here called Western Baltic (SD24), and east of Bornholm, here called Eastern Baltic (SD25–29; Figure 2).

The three areas are watersheds that differ hydrographically and ecologically: There is a gradient of increasing productivity and in salinity from the inner parts of the Baltic sea toward the oceanic conditions in eastern North Sea region (Andersen et al., 2015; Fonselius, 1995; Granéli et al., 1990). Cod in the Öresund is partly separated from other adjacent populations (Svedäng, André et al., 2010), and there is a clear‐cut genetic differentiation between Western and the Eastern Baltic stocks (e.g., Hemmer‐Hansen, Therkildsen, Meldrup, & Nielsen, 2014; Poćwierz‐Kotus et al., 2015). The areas also differ in exploitation patterns regarding gear selectivity, that is, the proportion between trawl and gill net fishing, as indicated below, and landings (Table S1). To this end, the vicinity of the different cod populations, each of them located in areas with different ecological settings and having different exploitation patterns and stock status, provides a case for investigating the roles of fishing regulation in comparison with ecosystem status for cod stock productivity, growth performance, and population stability.

Given these essential differences, the size structures in the three cod stocks are today different:

- The *Öresund cod* is renowned for a relatively high abundance of, in particular, larger individuals and moderate or at least sustainable exploitation rates (Lindegren et al., 2013; Sundelöf, Wennhage, & Svedäng, 2013; Svedäng, Stål, Sterner, & Cardinale, 2010). This situation is partly due to the resident behavior of this stock in the central Öresund (Svedäng, André et al., 2010), and, partly as a result of a ban on towed fishing gears, that is, gillnetters is dominating the cod fishing in most of the Öresund. This trawling ban was implemented already in 1932 (Anonymous, 1932), apart from a northern area adjacent to the Kattegat where trawling is still allowed, albeit restrictions have been put in place since 2009 also for this area (e.g., ICES, 2012). There are indications of a behavioral stock separation between spawning cod in this northern area close to the Kattegat and the central Öresund (Svedäng, André et al., 2010 and the references therein). The northern spawning unit in the Öresund is ephemeral in nature and seems only to be gathering in this area at spawning. There is no specific monitoring of this spawning unit and influences only the results of this study regarding the landings statistics from the Öresund.
- The *Western Baltic cod* is presently considered as overexploited. Trawl fishing dominates (60%). The stock increasingly mixes with the Eastern cod stock west of the Bornholm island (SD 24), especially over the past 10 years (Figure 2; Eero et al., 2015; ICES, 2016).
- The *Eastern Baltic cod* productivity has varied over time, partly because two out of three major spawning components/locations have almost disappeared (e.g., Cardinale & Svedäng, 2011). The Eastern Baltic cod is fished to ~85% with towed fishing gears and has had periods of both exceptional production levels in the 1980s and prolonged overfishing in the 1990s and 2000s. In recent years, better recruitment and reduced exploitation rates have forgone lower condition and poorer growth (Eero et al., 2015 and the references therein). Today, due to increasing difficulties in aging (Eero et al., 2015), the Eastern Baltic cod stock is regarded as data poor since 2014 (ICES, 2014) and the status is uncertain.

### 1.4 Aim of the study

There is an urgent interest of better understanding of the role of fishing compared to ecosystem status for Eastern Baltic cod productivity, and, in particular, the influence of trawl selectivity on productivity. The aim of this study was hence to investigate whether fishery‐independent catch rates alone can be used as a novel way of interpreting monitoring information together with catch data in a simplified yet more informative way to elucidate changes in growth and productivity.

## 2 MATERIALS AND METHODS

### 2.1 Data structure

In this study, we present trawl survey data from the Baltic Sea (SD24–29) and the central Öresund (SD23) between 1991 and 2016. Details on monitoring fishing procedures, carried out by nine countries having a random stratified design, are given by ICES (2013a). We argue that this naturally occurring stock set up may be used for forwarding novel ways of assessing the present status of Baltic cod stocks.

The distinction between the central and northern Öresund is important for three reasons: (1) the central Öresund harbors a resident stock of cod, whereas in the northern administrative part of the Öresund next to the Kattegat cod gather at spawning (Svedäng, André et al., 2010 and the references therein); (2) a trawling ban has been implemented for the central Öresund since 1932 (Anonymous, 1932), whereas trawling has been allowed in the northernmost part unrestrictedly until recently (ICES, 2012); (3) monitoring fishing has been designated to follow the cod in the central Öresund, whereas the northern part has been overlooked (cf. ICES, 2012). Studies on cod population dynamics are all designated to this central part of the Öresund (Lindegren et al., 2013; Sundelöf et al., 2013; Svedäng, Stål et al., 2010).

We retrieved observations on the size structure of the Baltic cod stocks from the ICES DATRAS database (www.ices.dk; 1991–2016) for all those national surveys using the BITS (Baltic International Trawl Survey) program and standards (ICES, 2011). BITS surveys are carried out in the first and fourth quarter of the year (Q1 and Q4), with some exceptions at the beginning of the 1990s, using a TW3‐trawl. Table 1 shows the decadal mean number of hauls per quarter of the year. Albeit we do not use age data in this study, the similarity in gear selectivity and catchability over time for the standardized BITS surveys permits a more detailed analysis to be conducted, including information on CPUE, that is, number per length group per trawling hour. Noteworthy, surveys in the Öresund are comprised of a low number of hauls (Table 1), limiting the statistical power of this part of the study.

**Table 1:** The number of hauls and measured fish by decade for surveys in the first and fourth quarter for Eastern Baltic cod, Western Baltic, and the Öresund cod

Decade	Eastern Baltic cod		Western Baltic cod		The Öresund cod	
	Number of hauls	Number of fish	Number of hauls	Number of fish	Number of hauls	Number of fish
1990s						
Q1	2,205	786,314	398	259,313	12	12,264
Q4	299	78,004	287	2,377,864	3	1,275
2000s						
Q1	2,061	1,096,245	471	386,695	26	19,442
Q4	1,288	844,209	450	374,712	29	3,783
2010–2016						
Q1	1,306	1,396,441	354	318,767	23	23,930
Q4	879	849,541	252	184,860	19	3,249

Within the distribution area for each stock, catch per unit effort CPUE are estimated by including all valid hauls. Eastern Baltic cod is defined as catches from ICES SD25–29, the Western Baltic cod from SD24 and the Öresund cod from SD23, respectively (Figure 2). The mean CPUE per year and quarter for each stock is estimated as the total number of fish caught per length group divided by the number of all valid hauls. Furthermore, length observations were pooled by decade to minimize variability and standardize data. The obtained relative size distribution may fluctuate due to variation in recruitment, mortality, and growth.

As to compare growth estimates based on age determinations with the growth indicators presented in this study, we used previously made estimates of the von Bertalanffy growth parameter, *L*_∞*i*_ of *i* year class from the Eastern Baltic cod stock (Svedäng & Hornborg, 2014). These estimates are forward‐shifted 2 years (i.e., *i + *2), as *L*_*∞i*_ for year class *i* seem to be determined by the prevailing growth conditions at age 2.

### 2.2 Productivity estimations

Fish stock net instantaneous productivity, $\dot{P}$, is defined by the following equation (Quinn & Deriso, 1999, pp. 50–53):

(1) $$\dot{P}\;=\;\dot{B}\;+\;\dot{Y}$$

where $\dot{B}$ is the instantaneous change in biomass and $\dot{Y}$ is the instantaneous change in yield. The annual surplus production is thus the integral of productivity. The equilibrium yield through harvesting can be regarded as the surplus production. In this study, we define biomass in relative terms, using catch per unit effort in weight (CPUE_*W*_), as a proxy on the biomass of the stock.

Sinclair (1998) suggests that a ratio between commercial catches, TC*,* and an index of abundance such as CPUE_*W*_, describes the exploitation rate, *R*, in a fish stock. This parameter *R* would thus function as a proxy on fishing mortality, *F*. However, such an interpretation is only possible by assuming constant recruitment, individual growth rate, and natural mortality rate, *M*. On the other hand, Mertz and Myers (1998) suggested that TC of piscivorous species reflects available production, *P*, as most of the available production is harvested for large, long‐lived species. Variation in production levels would thus for such species depend on growth, mortality rates, and the number of incoming recruits.

In other words, variation in catch level may reflect variation in *R* as well as in stock productivity, *P/B*. As the TAC (Total Allowable Catch) has seldom been restrictive for Eastern Baltic cod (ICES, 2015), TC, which includes estimates of discards, is in this study considered as a valid estimate of *P* in the stocks. We, therefore, interpret the ratio TC*/*CPUE_*W*_ as a proxy for *P/B* rather than as *R* in our study. This conjecture is supported by the fact that fishing pressure has remained high in the Baltic (*F*_4–6_ ≈ 1; Svedäng & Hornborg, 2014), as well as in the Öresund (*F*_4–7_ = 1.06; Lindegren et al., 2013), whereas the level of growth and recruitment determines the productivity of the stock.

### 2.3 Method for estimating growth indicators for trawl surveys

The length distributions are divided into 1 cm length classes (LC). Mean abundance by year, *j,* and, quarter of the year, *q,* in survey catch ($N_{jq}$) is the summed mean CPUE ($N_{\mathrm{LC}}$) for all occupied length classes (Probst, Stelzenmüller, & Kraus, 2013, and the references therein):

$$N_{jq}=\sum_{k=L_{\min}}^{L_{\max}}N_{\mathrm{LC}}$$

Mean length (ML_*jq*_) is estimated by the following equation (Probst et al., 2013, and the references therein):

$${\text{ML}}_{jq}=\sum_{k=L_{\min}}^{L_{\max}}{\text{LC}}_{k}\frac{N_{\mathrm{LC}}}{N_{jq}}$$

Mean individual weight in the catch, ${\text{MW}}_{jq},$ is estimated using the weight‐length relationship, where *a* = 0.104 and *b* = 3; (Cardinale & Hjelm, 2012):

$${\text{MW}}_{jq}=a{\mathrm{ML}}_{jq}^{b}$$

Length class richness, LCR_*jq*_, is defined as the total number of occupied of LC (Probst et al., 2013, and the references therein):

$${\text{LCR}}_{jq}=-\sum_{k=L_{\min}}^{L_{\max}}{\text{LC}}_{k}$$

Analysis of LCR against time is shown as supporting material.

Length class diversity, LCD_*jq*_, is defined by the Shannon–Wiener diversity of the annual length‐frequency distribution (Probst et al., 2013, and the references therein):

$${\text{LCD}}_{jq}=-\sum_{k=L_{\min}}^{L_{\max}}\frac{N_{\mathrm{LC}}}{N_{jq}}\text{ln}\left(\frac{N_{\mathrm{LC}}}{N_{jq}}\right)$$

Mean weight per length class (MWPLC_j*q*_): Total annual mean weight in the catch, TW_*jq*_, is divided with the number of occupied length classes, *N*_LC_, in survey catches by year and quarter of the year:

$${\text{MWPLC}}_{jq}={\text{TW}}_{jq}/{\text{LCR}}_{jq}$$

The productivity of the different stocks equals production‐biomass ratios, *P/B*. It is suggested that estimated total catches (TC) by stock (ICES, 2016; Table S1) represent stock production *P* and biomass of the stock, *B*, is given by the product of mean annual individual weight in the catch, *MW*_*j*_, and $N_{j}$ in the first quarter of the year:

$$\frac{P_{j}}{B_{j}}=\text{TC}/\left({\text{MW}}_{j}*N_{j}\right)$$

The *P/B*‐ratio is normalized for each stock to the proportion of the maximum observed value for this parameter.

### 2.4 Statistical analysis

The correlations between different parameter values by year, *j,* and, the quarter of the year, *q,* are evaluated by estimating Spearman's rho using the r‐script package “corrgram version 1.10” (Wright, 2016). We also studied the linear relationships between various parameters over time are investigated using a linear function in the r‐script package “lme4” (Bates, Maechler, Bolker, & Walker, 2015). We assume that *p > *.05 indicate nonsignificant results.

## 3 RESULTS

### 3.1 Cod in the Eastern Baltic (SD25–29)

**Figure 3:**
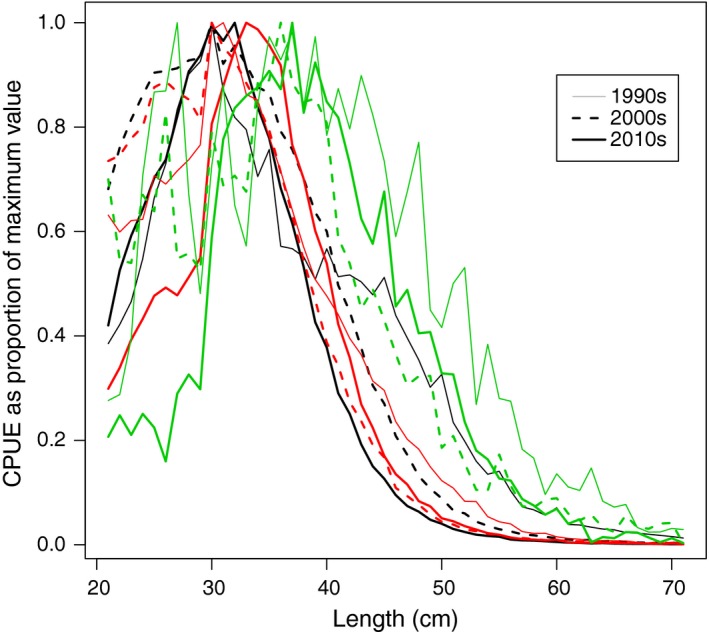
Normalized size distribution as the proportion of maximum observed CPUE for cod of sizes between 20 and 70 cm per decade for Eastern Baltic 1990s–2010s (in black); Western Baltic 1990s–2010s (in red); Öresund 1990s–2010s (in green)

**Figure 4:**
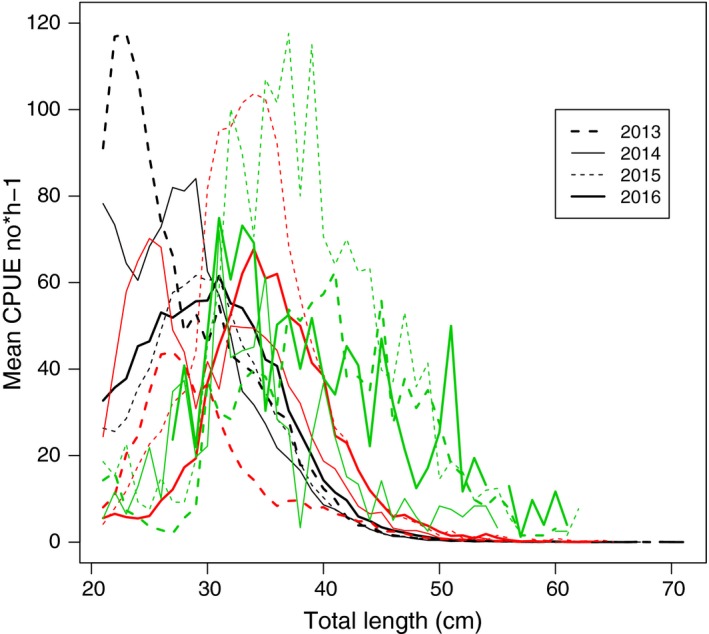
CPUE for cod by length group between 20 and 70 cm by quarter 1 between 2013 and 2016 for (a) Eastern Baltic (in black); (b) Western Baltic (in red); (c) Öresund (in green)

Collated length distributions show that the Eastern Baltic cod stock has gone from a wider to a truncated size distribution since the 1990s (Figures 3 and S1). The initially rather high abundances of fish in larger length groups decline over time, where the present size distribution is the most extreme with most fish found around 30 cm (Figure 4).

**Figure 5:**
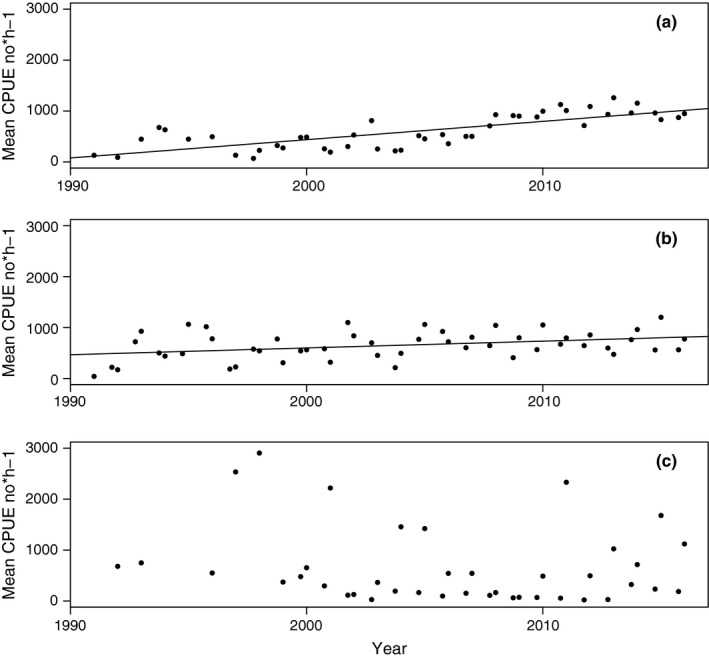
Mean CPUE for cod in all length classes ≥20 cm between 1991 and 2015 by survey occasion (quarter 1 and 4) for (a) Eastern Baltic; (b) Western Baltic; (c) Öresund. Slope of linear regression is shown if *p *<* *.05 of the regression coefficient

**Figure 6:**
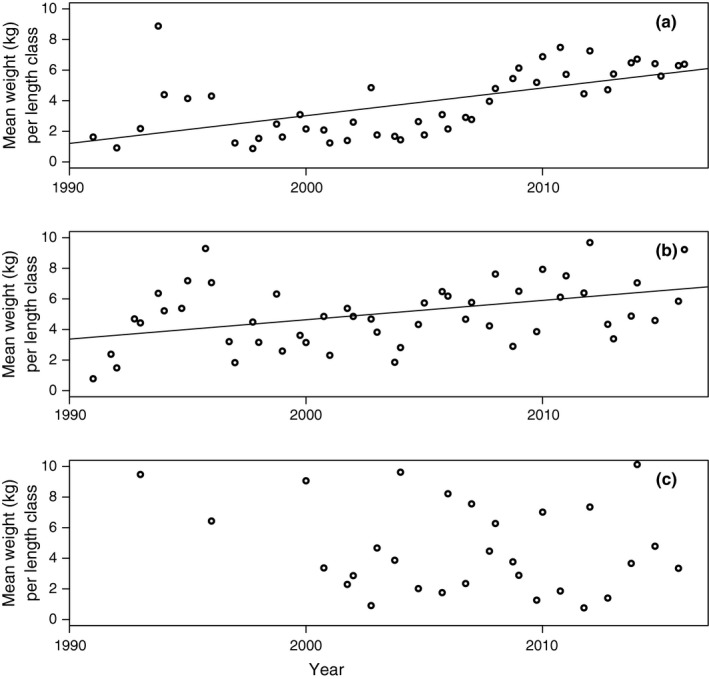
Mean weight per length class (MWPLC, kg per cm length group) for length groups with presence cod ≥20 cm 1991–2015 by survey occasion (quarter 1 and 4) for (a) Eastern Baltic; (b) Western Baltic; (c) Öresund. Slope of linear regression is shown if *p *<* *.05 of the regression coefficient

**Figure 7:**
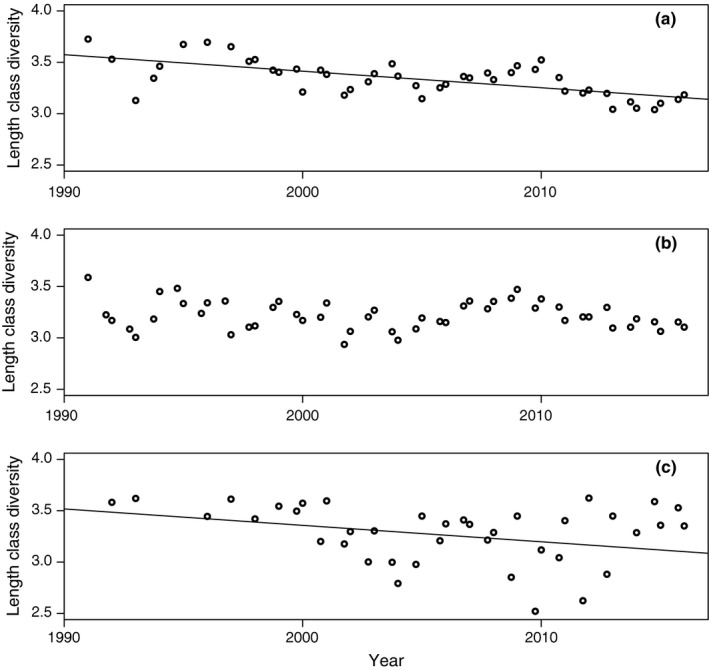
Length class diversity (LCD) in the catch for cod with ≥20 cm in total length 1991–2015 by survey occasion (quarter 1 and 4) for (a) Eastern Baltic; (b) Western Baltic; (c) Öresund. Slope of linear regression is shown if *p *<* *.05 of the regression coefficient

**Figure 8:**
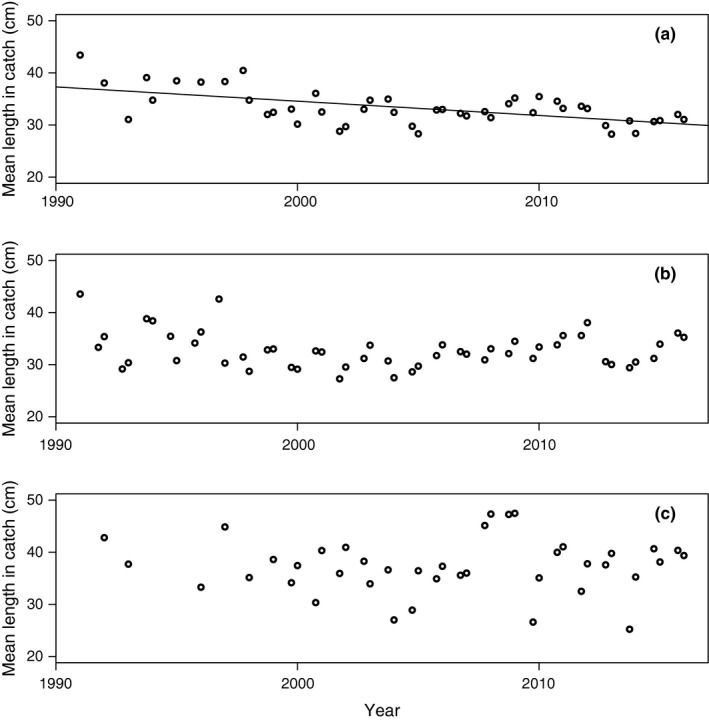
Mean individual length (ML, in cm) in the catch for cod with ≥20 cm in total length 1991–2015 by survey occasion (quarter 1 and 4) for (a) Eastern Baltic; (b) Western Baltic; (c) Öresund. Slope of linear regression is shown if *p *<* *.05 of the regression coefficient

Table 2 shows correlation matrix (Spearman's rho and corresponding *p*‐values) for various growth and size indicators between 1991 and 2016. It is observed that the mean CPUE $\left(N_{jq}\right)$ increases over time with a peak at the beginning of the 2010s (Table 2, Figure 5a; Table S2). In accordance, MWPLC increases over time (Table 2, Figure 6a; Table S3) and is positively correlated to CPUE (Table 2), as fish become concentrated to fewer length groups. Consistently, there are negative correlations between *Year* and LCD (Table 2, Figure 7a; Table S4). Accordingly, LCD is negatively correlated with CPUE and MWPLC (Table 2), which implies that growth and length class diversity deteriorate at higher abundances of cod. There is a positive correlation between LCD and ML, showing an expected degree of collinearity between some variables. ML declines consistently over time and has a negative, however weak, correlation with CPUE but not to MWPLC (Table 2; Figure 8a and Table S5). The von Bertalanffy's growth factor, *L*_*∞*_, correlates negatively to *Year* (described in Svedäng & Hornborg, 2014), is also negatively correlated to ML and, in particular, with LCD (Table 2). These three growth indicators are rather closely related. However, changes in *L*_*∞*_ are not significantly correlated to CPUE or MWPLC.

**Table 2:** Correlation matrix (Spearman's rho and corresponding *p*‐values) of the time and various growth indicators for the upper panel: Eastern Baltic cod, middle panel: Western Baltic cod, lower panel: The Öresund cod

*p*	Rho					*L*_∞_
	Year	Catch per unit effort (CPUE)	Mean weight per length group (MWPLC)	Mean length in catch (ML)	Length class diversity (LCD)	
*Eastern Baltic cod (SD25–29)*						
Year	1	0.79	0.67	−0.49	−0.64	−0.62
CPUE	<0.0001	1	0.93	−0.37	−0.55	−0.44
MWPLC	<0.0001	<0.0001	1	−0.14	−0.40	−0.26
ML	0.0005	0.0109	0.3528	1	0.8	0.54
LCD	<0.0001	<0.0001	0.0054	<0.0001	1	0.75
*L*_∞_	0.0050	0.0570	0.2898	0.0182	0.0002	1
*Western Baltic cod (SD24)*						
Year	1	0.34	0.38	−0.05	−0.12	
CPUE	0.0141	1	0.81	−0.15	−0.14	
MWPLC	0.0058	<0.0001	1	0.35	0.09	
ML	0.7315	0.3052	0.0119	1	0.54	
LCD	0.3906	0.3380	0.5318	<0.0001	1	
*The Öresund cod (SD23)*						
Year	1	−0.20	−0.11	0.11	−0.29	
CPUE	0.2160	1	0.95	0.01	0.56	
MWPLC	0.4992	<0.0001	1	0.24	0.6	
ML	0.5046	0.9377	0.1445	1	0.43	
LCD	0.0731	0.0002	<0.0001	0.0057	1	

Dark gray: *p *<* *.01, light gray: *p *<* *.05, white: NS. *L*_∞_ is a von Bertalanffy growth parameter of year class *i* of the Eastern Baltic cod and it is forward‐shifted 2 years (from Svedäng & Hornborg, 2014).

**Figure 9:**
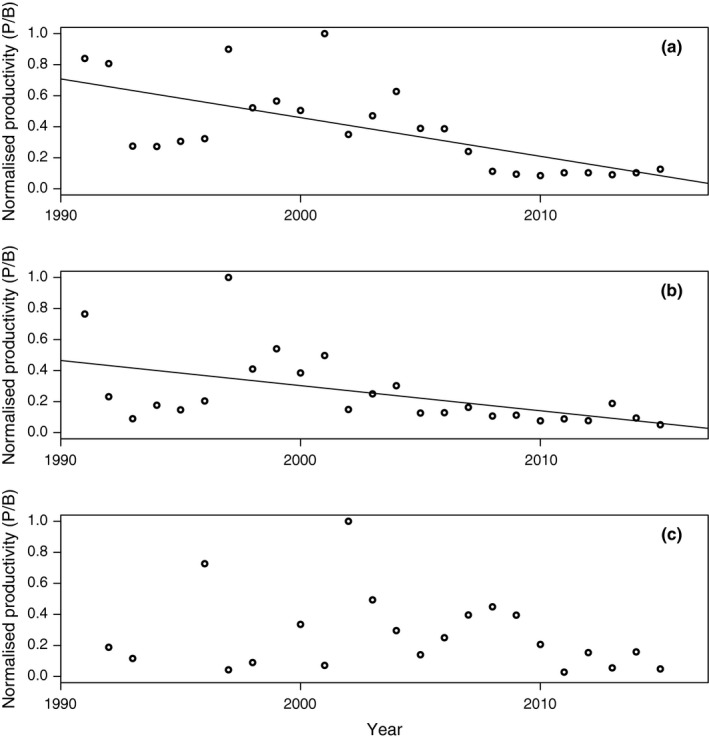
The normalized productivity index (*P/B*) 1991–2015 based on the ratio between yearly landings and estimates of mean catch biomass in the first quarter of the year for (a) Eastern Baltic; (b) Western Baltic; (c) Öresund). Slope of linear regression is shown if *p *<* *.05 of the regression coefficient

The productivity of the Eastern Baltic cod stock, *P/B*, is considerably reduced over the studied period (Figure 9a; Table S6). Noteworthy, this decline occurred in spite of a concomitant reduction in ML (or mean weight), because a lower denominator would be expected to increase the value of the ratio. This decrease in *P/B* is thus partly due to a lowered catch level (Table S1) and partly related to increases in CPUE, that is, a more abundant stock, strongly suggesting lower productivity due to reduced individual growth.

### 3.2 Cod in the Western Baltic (SD24)

For fish larger than 30 cm, Western Baltic cod are more truncated in the 1990s and 2000s than the Eastern Baltic cod, whereas in the 2010s the relationship is the opposite, where the proportion of fish larger than 40 cm is higher for Western than for Eastern Baltic cod (Figures 3, 4; Figure S1).

Mean CPUE ($N_{jq})$for fish >20 cm shows a positive significant relationship over the studied time 1991–2016 (Table 2; Figure 5b; Table S2). In similarity to the Eastern Baltic cod, there is a strong positive trend in MWPLC over time (Table 2; Figure 6b; Table S3). No trend can be detected over time in LCD (Table 2, Figure 7b; Table S4), showing that a similar set of length groups in Western Baltic cod have become more abundant over time. It is no change in ML over time (Table 2, Figure 8b; Table S5). Increased abundance has not led to increased size diversity, as ML is not correlated to CPUE*,* neither is LCD correlated to CPUE nor MWPLC, whereas MWPLC and ML are positively correlated (Table 2). In similarity to Eastern Baltic cod, there are positive correlations between LCD and ML*,* MWPLC and CPUE, indicating collinearity between some variables.

The productivity, measured as *P/B,* of the Western Baltic cod, decreases in parallel to the Eastern Baltic cod, albeit it exhibits a somewhat higher inter‐annual variation (Figure 9; Table S6). This palpable reduction in *P/B* is to a large extent related a decline in catches (Table S1).

### 3.3 Cod in the Öresund (SD23)

The size distribution of the Öresund cod stock shows no trend toward becoming wider or more truncated over the studied last 25 years (Figures 3; Figure S1). The broadest size distribution, that is, with highest occurrence of large‐sized cod, is encountered in the 1990s, albeit the results from the 1990s are based on relatively few hauls (Table 1). In the 2000s, the size distribution twists toward small‐sized cod, whereas larger length groups become more abundant again in the 2010s. From 2013 Q1 and onwards, small‐sized cod (<30 cm) become more scarce, whereas the occurrence of cod >30 shows large variation between years (Figure 4).

Mean CPUE for fish >20 cm shows considerable variation between separate survey occasions, but there is no trend over the time 1991–2016 (Table 2, Figure 5c; Table S2). This high variance is consistent with the low sample size (low number of hauls) of the survey in this area (Table 1). It should be observed that cod abundance (CPUE) is occasionally much higher in the Öresund compared to the Western and Eastern Baltic cod stocks (Figure 5a–c).

There is no significant trend over time in MWPLC (Table 2; Figure 6c; Table S3), whereas a weak negative trend is found in LCD (Table 2, Figure 7c; Table S4). Mean length, ML*,* is stable over time (Table 2, Figure 8c; Table S5) and shows no correlation to neither CPUE nor MWPLC, whereas CPUE and MWPLC are positively correlated. There are no indications of density dependence as there are only positive correlations between abundances and growth indices, that is, between CPUE and LCD as well as between LCD and MWPLC, and between LCD and ML (Table 2).

There is no trend in productivity of the Öresund cod stock (Figure 9; Table S6). The considerable reduction in *P/B* in recent years is almost entirely related to a decline in catches (Table S1), whereas neither ML nor CPUE show any trend.

## 4 DISCUSSION

### 4.1 Changes in size structure

This study reports marked differences in how the relative size structure of Baltic cod has developed from the 1990s to present; the Eastern and Western Baltic cod have gone toward more truncated size structures, most pronounced for the Eastern Baltic cod. Furthermore, this progression of increasingly contracted size distributions is inversely related to the stock biomass (cf. Eero et al., 2012). For the Öresund cod, on the other hand, there is no trend toward a more truncated size distribution over the study period but varied over time. Noteworthy, both as a share and abundance, large cod are much more common in the Öresund than the next Kattegat or North Sea (cf. Svedäng, Stål et al., 2010; Sundelöf et al., 2013).

Similar to the Baltic cod stocks, Shelton et al. (2006) reported that truncation in size structure and reduction in productivity followed depletion due to excessive fishing in many of the Northwest (NW) Atlantic cod stocks. Furthermore, the slow recovery of the NW Atlantic cod stocks is related both to poor recruitment and impaired growth (cf. Lilly et al., 2008; Morgan, Shelton, González‐Costas, & González‐Troncoso, 2016). In the Baltic cod stocks, on the other hand, relative recruitment measured as recruits per unit spawning biomass (*R/SSB*) has been much higher and *increasing* during the time when the indices of growth here shown started to decline (Eero et al., 2012). The recovery of the Baltic cod stocks is thus primarily hampered by factors related to the truncated size distribution.

### 4.2 Effects of fishing pressure

Earlier studies have suggested that increased fishing pressure in offshore areas may have changed the size distribution considerably in the Baltic Sea since the 1940s and onwards (Eero, Köster, & MacKenzie, 2008; Meyer, 1952; Zeller et al., 2011). Already between 1940 and 1943, a drastic decline occurred in the proportion of large‐sized fish in landings from the Eastern Baltic Sea (from 64% to 37%; Meyer, 1952), connected with a wartime relocation of German offshore trawl fishery from the North Sea to the Baltic Sea. Eero, Köster, Plikshs, and Thurow (2007) point out that the fishing mortality (*F*) was relatively moderate immediately after the Second World War, however, increased to ~1 already in the late 1950s. Inspections of landings of the Swedish Baltic cod trawl fishery in the 1960s corroborate this view (Otterlind, 1984); cod bigger than 50 cm in total length declined markedly. Analytical assessments are available since 1966, showing intense fishing pressure ever since, except for a reduction in *F* that has purportedly taken place in recent years (e.g., ICES, 2013b). On the other hand, re‐calculations of *F* in the Baltic Sea, considering unaccounted removals and discards, indicate fishing mortality to be over 30% higher than given by analytical assessments (Zeller et al., 2011). Although *F* might not have decreased as much as presently suggested (e.g., ICES, 2013b), the decline in fishing effort in recent years at least suggests that changes in size distribution are unlikely linked to an elevation in *F*.

### 4.3 Interplay between variation in ecosystem productivity and individual cod growth

Besides variation in recruitment and fishing mortality on different size classes, variation in individual growth is often of paramount importance for the shaping of the size distribution. This kind of variability has in particular been found important for areas such as the Baltic Sea, where fish growth often has been shown to be stunted (Eero et al., 2015; Hessle, 1933; Meyer, 1952). Our current understanding of Baltic cod productivity back in time relies on model predictions (Eero, MacKenzie, Köster, & Gislason, 2011). Drivers in these projections are climate variability, as it affects the periodicity in saltwater inflows to the Baltic (cf. Carstensen, Andersen, Gustafsson, & Conley, 2014), seal abundance, fishing intensity, and eutrophication levels. Thurow (1997) inferred that fish biomass was low at the beginning of the 1900s due to the overall low productivity of the Baltic Sea and the predatory pressure from abundant seal populations. With increasing eutrophication over the 20th century (Andersen et al., 2015; Conley et al., 2009) and depletion of top predators in early 1900s, more productive states have emerged for species such as cod, herring (*Clupea harengus*) and sprat (MacKenzie et al., 2002).

However, in contrast to such a narrative, inspection of the historical fishery and trading statistics from Baltic countries tells yet another story; cod biomass has fluctuated over the last 500 years, suggesting that cod biomass might periodically have exceeded present biomass levels (MacKenzie et al., 2007). Even further back in time, von Bertalanffy growth modeling and back calculation suggest that Neolithic Baltic cod grew to smaller asymptotic lengths, but were larger at younger ages, implying rapid early growth (Limburg et al., 2008).

Earlier studies have suggested that cod growth is density dependent in the Baltic Sea. Hessle (1933) observed that growth increased from the south to the north of the Baltic as cod abundance declined, noteworthy *against* the salinity gradient. Meyer (1952) suggested that length‐at‐age may have decreased in the stock because the large‐sized fish had disappeared due to fishing and small juvenile fish had become more numerous. These observations from the first part of the 20th century, together with more recent findings (Eero et al., 2012; Svedäng & Hornborg, 2014), may imply that cod growth often is hampered in the Baltic Sea. Density‐dependent factors, that is, ultimately intraspecific feeding competition, have been a recurrent phenomenon in the Baltic cod, possibly sprung from the specific environmental conditions of the semi‐enclosed, brackish sea. Given these natural conditions of the Baltic Sea, density dependence is thus more easily induced in cod stocks by aiming for too high a selectivity (cf. Beverton & Holt, 1957), as also indicated in this study.

### 4.4 Effects of size‐selective fishing on fish growth

This study confirms previous studies that the Öresund cod have a wider size structure and is probably much more productive than the other two Baltic cod stocks (ICES, 2015; Lindegren et al., 2013; Sundelöf et al., 2013; Svedäng, André et al., 2010; Svedäng, Stål, et al., 2010). A plausible explanation would be the use gill nets instead of trawls in the Öresund fishery, the latter being the dominating form of cod fishing in both the other areas, even if less so for the Western Baltic compared to the Eastern Baltic cod stock. The bell‐shaped type of selectivity in gill net fishing compared to trawl fishing, where the selectivity curve is very steep on the left‐hand side, while continuously flat on the right one (Harley & Myers, 2001), has in fact also earlier been suggested to stabilize population dynamics (Hutchings, 2009).

### 4.5 Combined effects of selectivity and ecosystem productivity on fish growth

The possibly lower ecosystem productivity in the Western and Eastern Baltic Seas could be extra detrimental for the individual growth when combined with an increase in trawl selectivity (cf. Beverton & Holt, 1957). The higher levels of abundance (CPUE) and weight per occupied length group (MWPLC) in Western and Eastern Baltic cod at the latter parts of the time series, may not contradict the notion of a lower ecosystem productivity in the Baltic sea compared to the Öresund. The low condition (Casini et al., 2016) and the decline of the growth indices for Western and Eastern Baltic cod (this study) rather confirm the feeding restrictions.

Failed recoveries of depleted fish stocks have before been attributed to selective fishing leading to permanent lower growth (e.g., Swain, Sinclair, & Hanson, 2007). Density dependence and evolutionary change may, in fact, work in concert (Eikeset et al., 2016). Noteworthy, contrary to trawl fishing, bell‐shaped selectivity patterns are believed to counteract fishing‐induced evolutionary changes in fish populations (Hutchings, 2009).

### 4.6 Changes in productivity and growth 1991–2016

Mertz and Myers (1998) noted that yield could approximate production in many piscivorous fish stocks. This procedure may be especially applicable in a situation where there are no restrictions on the amount of fishing (i.e., no effort regulation), and the TAC is not limiting. Interpreting lower landings of Eastern Baltic cod as lower exploitation rate instead of potentially lower growth can be erroneous (e.g., ICES, 2016). To our knowledge, the Eastern Baltic cod fishery is the only example where a severe lowering of the condition in a fish stock (e.g., Casini et al., 2016; Eero et al., 2015) is not considered to lead to impaired growth.

On the contrary, we suggest that lowered individual growth may explain the reduction in landings (recent years’ quota uptake not more than 40%–65%), lower condition factor, lower length class richness and diversity, and higher abundance of small‐sized fish as well as an increase in the mean weight per length class. We also observe that the time series on *L*_*∞*_ estimated in Svedäng and Hornborg (2014) correlate to length class diversity, further corroborating previous hypotheses on stunted growth (Eero et al., 2012; Svedäng & Hornborg, 2014). Furthermore, the reduced individual growth in the Eastern Baltic cod stock leads ultimately to a loss of productivity over the studied period.

In similarity to the Eastern Baltic cod stock, lower landings in the Western Baltic stock may indicate a decline in productivity. However, the Western Baltic cod are increasingly mixing with migrating fish from the Eastern cod stock (e.g., ICES, 2016). It is hence questionable to conclude that stunted growth also occurs to the same degree in the Western cod stock; the explanation might be that the Eastern cod that have expanded to the west into SD24 have a similar growth pattern.

In contrast to Eastern and Western Baltic cod stocks, the time series on the productivity of the Öresund cod stock shows no trend. The reduction in *P/B* in recent years is almost entirely related to a decline in catches, whereas ML and CPUE have been rather stable over time. This drop in landings is related to the restrictions that have been implemented on the trawl fishery in the first quarter of the year in the northernmost part of the Öresund since 2009, where previously trawlers were targeting spawning aggregations. This recent change in exploitation pattern has led to a decrease in the overall catch level in the Öresund with about 50% (ICES, 2012). Thus, there is no evidence at hand for suggesting a decline in individual growth in the central Öresund cod, as reduced fishing in the northernmost part of the Öresund may explain lower landings. Thus, the productivity level can be assumed to be more or less stable in most of the Öresund.

### 4.7 Implications for management of Eastern Baltic cod

Our results imply that the scope for the harvesting of Eastern Baltic cod might be much more limited than perceived in regular assessments (e.g., ICES, 2015, 2016). There is an urgent need for recognizing the present status of Eastern Baltic cod without having to rely on aging. According to the BITS survey, the number of cod around 30 cm in length has during the 2010s remained at very high levels. Over time, such amount of fish would have transformed into larger sizes unless mortality has been very high, or, as we argue, induction of density dependence that impairs growth (Svedäng & Hornborg, 2014).

Tentatively, an important observation is that of the similarities between current situation for Eastern Baltic cod to what accompanied the Northern cod (Newfoundland Grand Banks and Labrador cod) collapse in the 1990s (Rose & Rowe, 2015; and the references therein). In the Gulf of St. Lawrence, the combined effects of density, size‐selective fishing, and temperature have been suggested to cause a reduction in growth (Sinclair, Swain, & Hanson, 2002). In the case of Eastern Baltic cod, the results in this study illustrate striking similarities, but even more, severe reductions in growth without temperature changes.

### 4.8 General implications of the study

Unsustainable fishing may arise not only from high fishing pressure or high and fluctuating fishing pressure (cf. Jonzén et al., 2002; Anderson et al., 2008) but also from management actions intended to promote sustainability. Such actions often include increased size selectivity to minimize discards of juveniles, which may lead to lower productivity in parts of a size‐structured population (cf. Beverton & Holt, 1957). Interactions could appear between selectivity, individual growth performance and environmental constraints, for the latter in particular at high recruitment pulses or detrimental environmental changes as fall in water temperature (cf. Chouinard & Swain, 2002). Altered age and size structures will also influence the productivity of stock; fish in an earlier part of the life cycle grow faster than at the end of the life cycle (Holt, 2009).

To facilitate a more EBFM, we need to embrace a more realistic view on what ecosystems can produce regarding fish biomass. Our results indicate that strive for maximum sustainable yield (MSY) by adopting strategies of increased size selectivity (e.g., Froese, Stern‐Pirlot, Winker, & Gascuel, 2008; COM 134: http://eur-lex.europa.eu/legal-content/EN/TXT/?uri=COM:2016:134:FIN) may very well be contra productive. Modifications aiming at higher yields such as in the Baltic cod could instead result in ecosystem erosion, and lower than average harvests that would have been obtained by slightly lowering the short‐term objective for long‐term profitability and ecosystem function (cf. Larkin, 1977; Svedäng & Hornborg, 2015).

## 5 CONCLUSIONS

The changes in size distribution of the predominantly trawl‐fished Western and Eastern Baltic cod stocks is much more dramatic than for the nontrawled the Öresund cod. By applying a novel approach for assessing fish stocks, we find:

- Indices of stock status, growth, and productivity can be deducted using survey information without having to rely on aging,
- Lower ecosystem productivity may amplify the effects of selective fishing on cod growth in the Baltic Sea,
- As decline in growth indices co‐varied with higher abundance and increased mean weight per occupied length class, we demonstrate that fisheries‐induced density dependence may lay behind the impaired growth of Western and, in particular, Eastern Baltic cod stocks,
- We encourage the Baltic cod fisheries management to acknowledge that scope for harvesting is much more limited than perceived in regular assessments.
- Our results suggest that selective fishing may disrupt fish population dynamic stability. As a consequence, both the advisory and the management side need to embrace a more realistic view on what ecosystems can produce in terms of yield. Furthermore, as seen in the cod stocks in the Baltic, the type of selective pattern has also implications on productivity, where the bell‐shaped selectivity curve in gill net fishing has a less detrimental effect than trawling.
