# Supplementary material for: Historic changes in length distributions of three Baltic cod (Gadus morhua) stocks: Evidence of growth retardation
Source: Ecol Evol. 2017 Jun 28;7(16):6089–102. doi: 10.1002/ece3.3173 (PMC5574768; doi:10.1002/ece3.3173)
Supplement: Supplementary file 3 [file ECE3-7-6089-s003.docx]

| Year | Minimum landing size  (MLS)  (cm) | Landings of cod (tonnes) | | |
| --- | --- | --- | --- | --- |
|  |  | The Öresund (SD23) | Western Baltic (SD24) | Eastern Baltic (SD25-29) |
| 1965 |  |  | 17007 | 147352 |
| 1966 |  |  | 14587 | 186053 |
| 1967 |  |  | 15193 | 207179 |
| 1968 |  |  | 18970 | 226053 |
| 1969 |  |  | 13169 | 222814 |
| 1970 |  |  | 12596 | 206076 |
| 1971 |  |  | 14504 | 170266 |
| 1972 |  |  | 16092 | 152232 |
| 1973 |  |  | 16210 | 150655 |
| 1974 |  |  | 15245 | 155748 |
| 1975 |  |  | 12500 | 204255 |
| 1976 |  | 712 | 15353 | 207644 |
| 1977 |  | 1716 | 15079 | 167770 |
| 1978 |  | 1777 | 14603 | 163884 |
| 1979 |  | 2729 | 16290 | 242275 |
| 1980 |  | 3725 | 15366 | 356163 |
| 1981 |  | 2373 | 24933 | 337827 |
| 1982 |  | 1778 | 24775 | 327600 |
| 1983 |  | 1377 | 22750 | 343146 |
| 1984 |  | 1931 | 20506 | 400473 |
| 1985 | 33 | 1339 | 16757 | 323282 |
| 1986 | 33 | 975 | 13742 | 256406 |
| 1987 | 33 | 1640 | 14821 | 216421 |
| 1988 | 33 | 1276 | 18203 | 202040 |
| 1989 | 33 | 828 | 11950 | 182640 |
| 1990 | 33 | 842 | 11577 | 157733 |
| 1991 | 33 | 1663 | 7846 | 125258 |
| 1992 | 33 | 2739 | 5370 | 56786 |
| 1993 | 33 | 1275 | 7129 | 52269 |
| 1994 | 35 | 1628 | 13336 | 102812 |
| 1995 | 35 | 3158 | 13801 | 109590 |
| 1996 | 35 | 4031 | 23097 | 125632 |
| 1997 | 35 | 2663 | 18995 | 92062 |
| 1998 | 35 | 3074 | 16049 | 69727 |
| 1999 | 35 | 3521 | 18255 | 74833 |
| 2000 | 35 | 3149 | 16264 | 95308 |
| 2001 | 35 | 2817 | 16451 | 94219 |
| 2002 | 35 | 2409 | 9781 | 69202 |
| 2003 | 38 | 1925 | 13127 | 71501 |
| 2004 | 38 | 2320 | 9430 | 69779 |
| 2005 | 38 | 2621 | 10686 | 56702 |
| 2006 | 38 | 1914 | 10858 | 70175 |
| 2007 | 38 | 2731 | 13183 | 54989 |
| 2008 | 38 | 2139 | 12256 | 45980 |
| 2009 | 38 | 839 | 11259 | 51766 |
| 2010 | 38 | 1179 | 9016 | 53819 |
| 2011 | 38 | 1198 | 9641 | 54218 |
| 2012 | 38 | 1123 | 11053 | 58020 |
| 2013 | 38 | 960 | 7333 | 36375 |
| 2014 | 35 | 1361 | 7862 | 38536 |
| 2015 | 35 | 1232 | 7193 | 43670 |

Table S2. Statistics for the linear regression catch-per-unit-effort (cpue) on year (see also Figure 5).

Eastern Baltic cod: Linear regression of cpue on year:

Call: lm(formula = ebc_cpue ~ year)

Residuals:

Min 1Q Median 3Q Max

-354.41 -158.90 -6.55 185.49 464.10

Coefficients:

Estimate Std. Error t value Pr(>|t|)

(Intercept) -71388.862 9004.081 -7.929 5.11e-10 ***

year 35.913 4.492 7.996 4.09e-10 ***

Signif. codes: 0 ‘***’ 0.001 ‘**’ 0.01 ‘*’ 0.05 ‘.’ 0.1 ‘ ’ 1

Residual standard error: 214 on 44 degrees of freedom

(5 observations deleted due to missingness)

Multiple R-squared: 0.5923, Adjusted R-squared: 0.5831

F-statistic: 63.93 on 1 and 44 DF, p-value: 4.093e-10

Western Baltic cod: Linear regression of cpue on year:

Call: lm(formula = wbc_cpue ~ year)

Residuals:

Min 1Q Median 3Q Max

-439.70 -180.98 -29.57 184.16 529.03

Coefficients:

Estimate Std. Error t value Pr(>|t|)

(Intercept) -26034.641 9738.911 -2.673 0.01018 *

year 13.318 4.861 2.740 0.00855 **

Signif. codes: 0 ‘***’ 0.001 ‘**’ 0.01 ‘*’ 0.05 ‘.’ 0.1 ‘ ’ 1

Residual standard error: 255.5 on 49 degrees of freedom

Multiple R-squared: 0.1329, Adjusted R-squared: 0.1152

F-statistic: 7.507 on 1 and 49 DF, p-value: 0.00855

Öresund cod: Linear regression of cpue on year:

Call: lm(formula = sou_cpue ~ year)

Residuals:

Min 1Q Median 3Q Max

-695.13 -489.80 -304.04 53.48 2069.57

Coefficients:

Estimate Std. Error t value Pr(>|t|)

(Intercept) 47648.73 37550.14 1.269 0.212

year -23.43 18.72 -1.252 0.218

Residual standard error: 747.5 on 38 degrees of freedom

(11 observations deleted due to missingness)

Multiple R-squared: 0.0396, Adjusted R-squared: 0.01433

F-statistic: 1.567 on 1 and 38 DF, p-value: 0.2183

Table S3. Statistics for the linear regression mean weight per length group (mwlgr) on year (see also Figure 6).

Eastern Baltic cod: Linear regression of mwlgr on year:

Call: lm(formula = ebc_mwlgr ~ year)

Residuals:

Min 1Q Median 3Q Max

-2.3061 -1.2262 -0.2278 0.8104 6.9999

Coefficients:

Estimate Std. Error t value Pr(>|t|)

(Intercept) -359.45083 73.39502 -4.897 1.35e-05 ***

year 0.18124 0.03661 4.950 1.14e-05 ***

Signif. codes: 0 ‘***’ 0.001 ‘**’ 0.01 ‘*’ 0.05 ‘.’ 0.1 ‘ ’ 1

Residual standard error: 1.744 on 44 degrees of freedom

(5 observations deleted due to missingness)

Multiple R-squared: 0.3577, Adjusted R-squared: 0.3431

F-statistic: 24.5 on 1 and 44 DF, p-value: 1.137e-05

Western Baltic cod: Linear regression of mwlgr on year:

Call: lm(formula = wbc_mwlgr ~ year)

Residuals:

Min 1Q Median 3Q Max

-3.2629 -1.4977 0.1221 1.2207 5.1969

Coefficients:

Estimate Std. Error t value Pr(>|t|)

(Intercept) -248.43911 77.76529 -3.195 0.00245 **

year 0.12654 0.03881 3.260 0.00203 **

Signif. codes: 0 ‘***’ 0.001 ‘**’ 0.01 ‘*’ 0.05 ‘.’ 0.1 ‘ ’ 1

Residual standard error: 2.04 on 49 degrees of freedom

Multiple R-squared: 0.1783, Adjusted R-squared: 0.1615

F-statistic: 10.63 on 1 and 49 DF, p-value: 0.002028

Öresund cod: Linear regression of mwlgr on year:

Call: lm(formula = sou_mwlgr ~ year)

Residuals:

Min 1Q Median 3Q Max

-10.537 -7.264 -3.925 -1.097 33.582

Coefficients:

Estimate Std. Error t value Pr(>|t|)

(Intercept) 681.4356 615.5925 1.107 0.276

year -0.3345 0.3068 -1.090 0.283

Residual standard error: 11.88 on 36 degrees of freedom

(13 observations deleted due to missingness)

Multiple R-squared: 0.03197, Adjusted R-squared: 0.00508

F-statistic: 1.189 on 1 and 36 DF, p-value: 0.2828

Table S4. Statistics for the linear regression of length class diversity (lcdiv) on year (see also Figure 7).

Eastern Baltic cod: Linear regression of lcdiv on year:

Call: lm(formula = ebc_lcdiv ~ year)

Residuals:

Min 1Q Median 3Q Max

-0.39598 -0.06798 -0.01079 0.07597 0.27132

Coefficients:

Estimate Std. Error t value Pr(>|t|)

(Intercept) 35.580086 5.610226 6.342 1.06e-07 ***

year -0.016083 0.002799 -5.747 7.94e-07 ***

Signif. codes: 0 ‘***’ 0.001 ‘**’ 0.01 ‘*’ 0.05 ‘.’ 0.1 ‘ ’ 1

Residual standard error: 0.1333 on 44 degrees of freedom

(5 observations deleted due to missingness)

Multiple R-squared: 0.4288, Adjusted R-squared: 0.4158

F-statistic: 33.03 on 1 and 44 DF, p-value: 7.937e-07

Western Baltic cod: Linear regression of lcdiv on year:

Call: lm(formula = wbc_lcdiv ~ year)

Residuals:

Min 1Q Median 3Q Max

-0.29120 -0.08561 -0.01854 0.09912 0.33421

Coefficients:

Estimate Std. Error t value Pr(>|t|)

(Intercept) 8.687267 5.263148 1.651 0.105

year -0.002728 0.002627 -1.039 0.304

Residual standard error: 0.1381 on 49 degrees of freedom

Multiple R-squared: 0.02154, Adjusted R-squared: 0.001571

F-statistic: 1.079 on 1 and 49 DF, p-value: 0.3041

Öresund cod: Linear regression of lcdiv on year:

Call: lm(formula = sou_lcdiv ~ year)

Residuals:

Min 1Q Median 3Q Max

-0.73778 -0.14684 0.07781 0.20903 0.46584

Coefficients:

Estimate Std. Error t value Pr(>|t|)

(Intercept) 35.320911 14.975465 2.359 0.0236 *

year -0.015982 0.007465 -2.141 0.0388 *

Signif. codes: 0 ‘***’ 0.001 ‘**’ 0.01 ‘*’ 0.05 ‘.’ 0.1 ‘ ’ 1

Residual standard error: 0.2981 on 38 degrees of freedom

(11 observations deleted due to missingness)

Multiple R-squared: 0.1076, Adjusted R-squared: 0.08415

F-statistic: 4.583 on 1 and 38 DF, p-value: 0.03876

Table S5. Statistics for the linear regression of mean length (mlength) on year (see also Figure 8).

Eastern Baltic cod: Linear regression of mlength on year:

Call: lm(formula = ebc_mlength ~ year)

Residuals:

Min 1Q Median 3Q Max

-5.4255 -1.5682 0.0976 1.8437 6.3896

Coefficients:

Estimate Std. Error t value Pr(>|t|)

(Intercept) 582.47662 115.05539 5.063 7.84e-06 ***

year -0.27395 0.05739 -4.773 2.03e-05 ***

Signif. codes: 0 ‘***’ 0.001 ‘**’ 0.01 ‘*’ 0.05 ‘.’ 0.1 ‘ ’ 1

Residual standard error: 2.734 on 44 degrees of freedom

(5 observations deleted due to missingness)

Multiple R-squared: 0.3412, Adjusted R-squared: 0.3262

F-statistic: 22.78 on 1 and 44 DF, p-value: 2.032e-05

Western Baltic cod: Linear regression of mlength on year:

Call: lm(formula = wbc_mlength ~ year)

Residuals:

Min 1Q Median 3Q Max

-5.6236 -2.3302 -0.3641 1.8596 9.8464

Coefficients:

Estimate Std. Error t value Pr(>|t|)

(Intercept) 182.68126 130.49979 1.400 0.168

year -0.07481 0.06513 -1.149 0.256

Residual standard error: 3.424 on 49 degrees of freedom

Multiple R-squared: 0.02622, Adjusted R-squared: 0.006347

F-statistic: 1.319 on 1 and 49 DF, p-value: 0.2563

Öresund cod: Linear regression of mlength on year:

Call: lm(formula = sou_mlength ~ year)

Residuals:

Min 1Q Median 3Q Max

-12.1802 -2.3208 0.2033 2.9670 10.0842

Coefficients:

Estimate Std. Error t value Pr(>|t|)

(Intercept) 1.971e+01 2.706e+02 0.073 0.942

year 8.781e-03 1.349e-01 0.065 0.948

Residual standard error: 5.388 on 38 degrees of freedom

(11 observations deleted due to missingness)

Multiple R-squared: 0.0001115, Adjusted R-squared: -0.0262

F-statistic: 0.004237 on 1 and 38 DF, p-value: 0.9484

Table S6. Statistics for the linear regression of ratio between production and stock biomass (pb) on year (see also Figure 9).

Eastern Baltic cod: Linear regression of pb on year:

Call:lm(formula = ebc_pb ~ year)

Residuals:

Min 1Q Median 3Q Max

-0.35861 -0.12372 -0.00669 0.08207 0.56621

Coefficients:

Estimate Std. Error t value Pr(>|t|)

(Intercept) 50.321823 11.855333 4.245 0.000306 ***

year -0.024932 0.005919 -4.212 0.000332 ***

Signif. codes: 0 ‘***’ 0.001 ‘**’ 0.01 ‘*’ 0.05 ‘.’ 0.1 ‘ ’ 1

Residual standard error: 0.2134 on 23 degrees of freedom

(26 observations deleted due to missingness)

Multiple R-squared: 0.4355, Adjusted R-squared: 0.4109

F-statistic: 17.74 on 1 and 23 DF, p-value: 0.0003317

Western Baltic cod: Linear regression of pb on year:

Call: lm(formula = wbc_pb ~ year)

Residuals:

Min 1Q Median 3Q Max

-0.32584 -0.09552 -0.03142 0.07547 0.64852

Coefficients:

Estimate Std. Error t value Pr(>|t|)

(Intercept) 32.692330 11.368895 2.876 0.00854 **

year -0.016195 0.005676 -2.853 0.00900 **

Signif. codes: 0 ‘***’ 0.001 ‘**’ 0.01 ‘*’ 0.05 ‘.’ 0.1 ‘ ’ 1

Residual standard error: 0.2046 on 23 degrees of freedom

(26 observations deleted due to missingness)

Multiple R-squared: 0.2614, Adjusted R-squared: 0.2293

F-statistic: 8.141 on 1 and 23 DF, p-value: 0.008996

Öresund cod: Linear regression of pb on year:

Call: lm(formula = sou_pb ~ year)

Residuals:

Min 1Q Median 3Q Max

-0.2784 -0.1686 -0.0438 0.1439 0.7138

Coefficients:

Estimate Std. Error t value Pr(>|t|)

(Intercept) 14.246832 16.202903 0.879 0.390

year -0.006973 0.008083 -0.863 0.399

Residual standard error: 0.2482 on 19 degrees of freedom

(30 observations deleted due to missingness)

Multiple R-squared: 0.0377, Adjusted R-squared: -0.01295

F-statistic: 0.7443 on 1 and 19 DF, p-value: 0.399

Table S7. Statistics for the linear regression length class richness (LCR) on year (see also Figure S2).

Eastern Baltic cod: Linear regression length class richness on year:

Call: lm(formula = ebc_lcr ~ year)

Residuals:

Min 1Q Median 3Q Max

-27.6745 -4.0571 0.8507 5.9754 15.7030

Coefficients:

Estimate Std. Error t value Pr(>|t|)

(Intercept) 1722.9988 382.5927 4.503 4.87e-05 ***

year -0.8232 0.1909 -4.313 8.95e-05 ***

Signif. codes: 0 ‘***’ 0.001 ‘**’ 0.01 ‘*’ 0.05 ‘.’ 0.1 ‘ ’ 1

Residual standard error: 9.092 on 44 degrees of freedom

(5 observations deleted due to missingness)

Multiple R-squared: 0.2972, Adjusted R-squared: 0.2812

F-statistic: 18.61 on 1 and 44 DF, p-value: 8.945e-05

Western Baltic cod: Linear regression length class richness on year:

Call: lm(formula = wbc_lcr ~ year)

Residuals:

Min 1Q Median 3Q Max

-13.6316 -3.6725 -0.6613 3.5422 16.6290

Coefficients:

Estimate Std. Error t value Pr(>|t|)

(Intercept) 472.7911 228.7394 2.067 0.044 *

year -0.2084 0.1142 -1.826 0.074 .

Signif. codes: 0 ‘***’ 0.001 ‘**’ 0.01 ‘*’ 0.05 ‘.’ 0.1 ‘ ’ 1

Residual standard error: 6.001 on 49 degrees of freedom

Multiple R-squared: 0.0637, Adjusted R-squared: 0.04459

F-statistic: 3.334 on 1 and 49 DF, p-value: 0.07397

Öresund cod: Linear regression length class richness on year:

Call: lm(formula = sou_lcr ~ year)

Residuals:

Min 1Q Median 3Q Max

-18.9788 -8.1701 0.4594 7.3471 18.3177

Coefficients:

Estimate Std. Error t value Pr(>|t|)

(Intercept) 1476.6004 539.9706 2.735 0.00944 **

year -0.7166 0.2692 -2.662 0.01131 *

Signif. codes: 0 ‘***’ 0.001 ‘**’ 0.01 ‘*’ 0.05 ‘.’ 0.1 ‘ ’ 1

Residual standard error: 10.75 on 38 degrees of freedom

(11 observations deleted due to missingness)

Multiple R-squared: 0.1572, Adjusted R-squared: 0.135

F-statistic: 7.088 on 1 and 38 DF, p-value: 0.0113
